# Treasurer’s Report for Financial Year (FY) 2013

**DOI:** 10.1093/molbev/msu272

**Published:** 2014-10-25

**Authors:** Aoife McLysaght

**Affiliations:** SMBE Treasurer


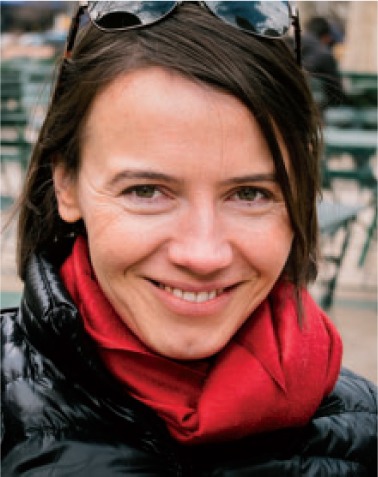


Society’s total assets as of May 31, 2014, are as follows: $2,933,887.35

• **Cash accounts** (community checking + money market business): $2,749,480.42

• **Total CDs**: $184,406.93

This report covers the period of June 1, 2013–May 31, 2014. Due to the ongoing success of *Molecular Biology and Evolution* (*MBE*) and *Genome Biology and Evolution* (*GBE*), whose revenues continue to increase, the society remains in outstanding financial health. Society membership decreased marginally during financial year 2013: Membership-only subscriptions decreased from 732 in 2012 to 332 in 2013, whereas Society membership + journal subscriptions increased from 313 in 2012 to 687 in 2013.

The society used its income to publish its two journals, *MBE* and *GBE*, and to support its annual meeting, in 2013, held in Chicago, IL. This included providing increased graduate and postdoctoral travel awards, funding the Walter Fitch Prize Symposium (a cash prize for the winner and travel funds for all eight contestants), running the Undergraduate Diversity Mentoring Program (UDMP), subsidizing the provision of on-site childcare, supporting the travel and accommodation of Council members, and providing travel incentives for associate editors of *MBE* and *GBE*. We also supported three satellite meetings on important, focused and timely topics related to molecular evolutionary biology.

We continue to receive an annual donation of $2,000USD from Masatoshi Nei to support an honorarium for publication in *MBE* of the annual Nei Lecture given by the President of Society for *MBE* (SMBE) during the annual meeting. The 2013 Nei Lecture is to be the first lecture published under this scheme. The total value of the honorarium is to be $4,000USD.

**Table:** 

Expense Category	June 1, 2013– May 31, 2014 (USD)
Interest income	532.23
OUP signing bonus (new contract negotiation)	125,000.00
OUP journal revenue (*MBE*)	585,510.00
OUP journal revenue (*GBE*)	131,224.00
Society fees collected by OUP	9,817.00
Annual meeting reimbursement	0.00
Nei Lecture fund donation (Masatoshi Nei)	2,000.00
*Total inflows*	*854,083.23*
2013 annual meeting	146,120.00
SMBE satellite meetings	54,796.50
Support for diversity programs (e.g., UDMP, childcare subsidy)	27,685.84
Nei Lecture publication honorarium	0.00
Bank charges	295.00
Accounting	1,750.00
Consultancy and professional society management	3,620.00
Corporation fee	40.00
Fitch prize	1,500.00
SMBE council and editors-in-chief travel	26,915.29
*MBE*/*GBE* associate editors travel incentive	110,001.07
SMBE travel awards (Fitch, graduate, postdoc)	46,969.00
SMBE website maintenance	514.81
SMBE treasurer expenses	0.00
*MBE* editorial office expenses	65,356.66
*MBE* editor expenses	66,000.00
*MBE* highlights and press releases	6,370.00
*GBE* editor expenses	0.00
*GBE* “Highlight” honoraria	10,090.50
Postage	0.00
*Total outflows*	*568,024.67*
*Overall total*	*286,058.56*

